# Biosolar cells: global artificial photosynthesis needs responsive matrices with quantum coherent kinetic control for high yield

**DOI:** 10.1098/rsfs.2015.0014

**Published:** 2015-06-06

**Authors:** R. L. Purchase, H. J. M. de Groot

**Affiliations:** Biophysical Organic Chemistry/Solid State NMR, Leiden Institute of Chemistry, PO Box 9502, 2300 RA Leiden, The Netherlands

**Keywords:** artificial photosynthesis, charge separation, solar fuel, responsive matrix, non-adiabatic coupling, quantum biology

## Abstract

This contribution discusses why we should consider developing artificial photosynthesis with the tandem approach followed by the Dutch BioSolar Cells consortium, a current operational paradigm for a global artificial photosynthesis project. We weigh the advantages and disadvantages of a tandem converter against other approaches, including biomass. Owing to the low density of solar energy per unit area, artificial photosynthetic systems must operate at high efficiency to minimize the land (or sea) area required. In particular, tandem converters are a much better option than biomass for densely populated countries and use two photons per electron extracted from water as the raw material into chemical conversion to hydrogen, or carbon-based fuel when CO_2_ is also used. For the average total light sum of 40 mol m^−2^ d^−1^ for The Netherlands, the upper limits are many tons of hydrogen or carbon-based fuel per hectare per year. A principal challenge is to forge materials for quantitative conversion of photons to chemical products within the physical limitation of an internal potential of *ca* 2.9 V. When going from electric charge in the tandem to hydrogen and back to electricity, only the energy equivalent to 1.23 V can be stored in the fuel and regained. A critical step is then to learn from nature how to use the remaining difference of *ca* 1.7 V effectively by triple use of one overpotential for preventing recombination, kinetic stabilization of catalytic intermediates and finally generating targeted heat for the release of oxygen. Probably the only way to achieve this is by using bioinspired responsive matrices that have quantum–classical pathways for a coherent conversion of photons to fuels, similar to what has been achieved by natural selection in evolution. In appendix A for the expert, we derive a propagator that describes how catalytic reactions can proceed coherently by a convergence of time scales of quantum electron dynamics and classical nuclear dynamics. We propose that synergy gains by such processes form a basis for further progress towards high efficiency and yield for a global project on artificial photosynthesis. Finally, we look at artificial photosynthesis research in The Netherlands and use this as an example of how an interdisciplinary approach is beneficial to artificial photosynthesis research. We conclude with some of the potential societal consequences of a large-scale roll out of artificial photosynthesis.

## 1. Why we need sustainable energy sources

Today, fossil fuels account for approximately 80% of the world's energy supply [1]. However, these resources will dwindle in the foreseeable future. Also, burning fossil fuels leads to emissions of large quantities of carbon dioxide (CO_2_), which is one of the major greenhouse gases causing global warming. Furthermore, fossil fuels are not evenly distributed around the world, leading to political tensions and potential problems with energy supply in countries that rely on imported fossil fuel. These arguments make sustainable, low carbon energy supplies one of the most pressing challenges facing society.

There are ample renewable energy sources on the planet for supplying mankind's increasing demand. The largest of these sources is solar energy. The conversion of energy from the Sun is therefore an obvious place to turn to when seeking alternative energy sources. There are a number of technologies for converting sunlight into electricity; the most common being photovoltaic cells. However, electricity is not readily stored, which means that the production of electricity has to be balanced with the demand at night time or during the winter season and it is not a practical energy source for applications such as long-distance air and sea transport. Thus, rather than stopping at the light capturing and charge separation steps of photosynthesis, there is increasing drive to mimic the processes of photosynthesis for fuel production.

Despite the growing momentum of research in this field, artificial photosynthesis remains largely unknown in energy and climate change policy documents [2]. As well as providing a mechanism for bringing together scientists working on artificial photosynthesis, a global consortium on artificial photosynthesis may serve to raise the visibility of this subject [3]. The most compelling argument for a global artificial photosynthesis derives from the sheer size of the energy system. Any new energy technology needs a huge human and industrial capacity as well as a capital investment of approximately 1 trillion dollars to bring it to 1% of the world's energy mix [4]. Such an effort can only be deployed on a truly global level.

The yield of artificial photosynthesis relates to the surface and higher efficiency means less surface. In this paper, we indicate current directions in the development of artificial photosynthesis devices that would fit in a global initiative, and challenges that need to be overcome in forging responsive matrix materials for quantitative conversion of photons to chemical products with high efficiency. In addition, we show how the Dutch BioSolar Cells consortium can be considered as a template for a global consortium on artificial photosynthesis. In the end, we reflect on the role of a global consortium and potential societal consequences of artificial photosynthesis.

## 2. Photosynthesis

**Figure 1.**
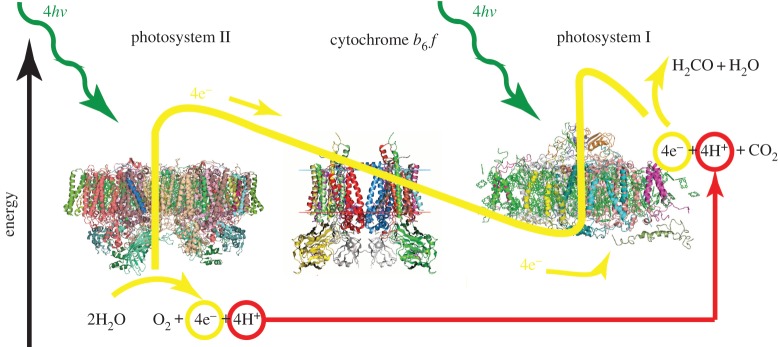
A schematic diagram of natural photosynthesis showing light absorption, charge separation, water oxidation and fuel production. The path of the yellow line indicates the approximate energy of the electrons in analogy to the *Z*-scheme. In recent years, progress has been made in elucidating the structures of many of the proteins involved in photosynthesis. This, in turn, helps us to understand and replicate their functions. (Online version in colour.)

Photosynthesis is the chemical process by which plants, algae and some bacteria store energy from the Sun in the form of carbohydrates that act as fuels. The four main steps of photosynthesis are light harvesting, charge separation, water oxidation and fuel production [5–7]. In light harvesting, antenna molecules, mostly chlorophyll but also carotenes, absorb sunlight and transfer the energy among themselves and eventually through to the reaction centre where charge separation takes place. In this way, energy from sunlight is used to separate positive and negative charges from each other. The positive charges are used to oxidize water. The electrons are transferred via cytochrome b_6_f and mobile electron carriers to photosystem I where they are excited again and used to produce carbohydrate fuel. A schematic diagram of what happens in photosynthesis is shown in figure 1.

The chemical reactions for water splitting and fuel production are:

(1) Water oxidation:
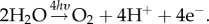
(2) Carbon dioxide reduction to produce carbohydrate fuel:
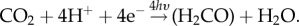

These two chemical half-reactions add up to give a total chemical reaction for photosynthesis of
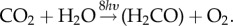

Four photons are required to drive each of the half reactions. Thus, eight photons are required for the total chemical reaction. As four electrons are carried over and eight photons are used, the process proceeds with two photons per electron. Nature uses two photosystems in tandem to drive the two chemical reactions of water splitting and fuel production. The reactions occur in proportion to the number of photons absorbed. Contrary to common belief, natural photosynthesis is not determined by insolation, the total amount of solar radiation energy that is collected per unit of time, but by the total light sum, the number of photons from the blue to red (400–700 nm) part of the spectrum that is collected per unit of time.

Although parts of the natural photosynthetic process are highly efficient, the overall solar-to-carbohydrate efficiency is low. Thus, unmodified natural photosynthesis cannot serve mankind's purposes for fuel production, but can be used as a blueprint for efficient artificial photosynthesis.

## 3. Artificial photosynthesis

The incoming photon flux, energy and electron transfer, and catalysis, operate on very different time, energy and length scales [8,9]. This puts design limits on how components should be matched for the most efficient solar-to-fuel conversion [8] operating close to the theoretical limits on solar energy conversion [10].

**Figure 2.**
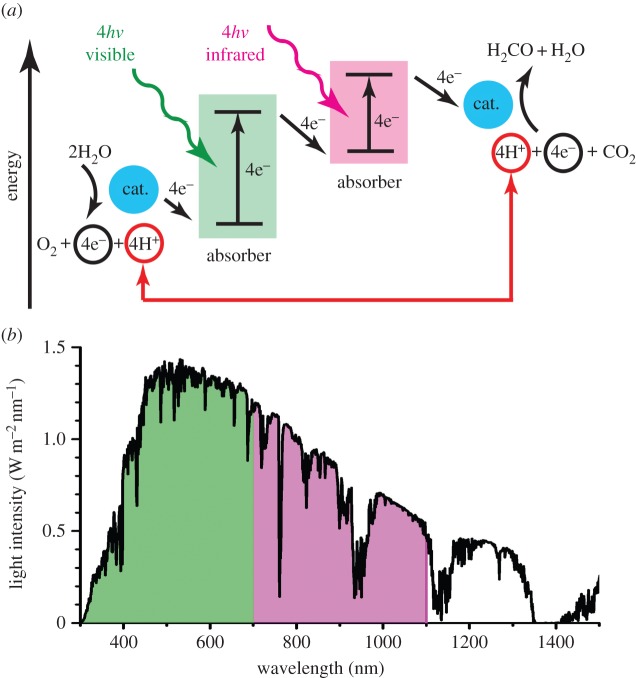
Schematic of a tandem artificial photosynthetic device (*a*) and its light-absorbing properties (*b*). This device operates in a fashion analogous to natural photosynthesis. As in figure 1, the approximate energy of the electrons as they pass through the device is shown. The tandem is in balance when both halves receive the same number of photons. An optimal use of the sunlight is reached with cut-off wavelengths of around 700 and 1100 nm. (Online version in colour.)

If we are going to make the best possible use of the incoming sunlight for fuel production with two photons per electron, it makes sense to capture as many photons of sunlight as possible. The photosynthetic apparatus in plants absorbs light around 700 nm. This means that plants use only half of the incoming photons. In comparison, silicon solar cells absorb light at around 1100 nm and therefore absorb more photons [11]. Nature uses two photosystems in tandem to drive the two chemical reactions of water oxidation and CO_2_ reduction. The same can be done with an artificial device. A weakness in the natural system is that the two photosystems absorb light of approximately the same energy, so the two systems are competing for the same photons while the infrared photons remain unused. In an artificial system, we can do this differently: have one absorber in the visible part of the spectrum and another in the infrared. In this way, the number of photons of sunlight that is absorbed by our system is maximized. Furthermore, the cut-off wavelengths are better matched to the electrochemical work [11]. Optimal matching is obtained with cut-off wavelengths of 700 and 1100 nm [12]. Thus, many researchers are now aiming to produce tandem devices that have two absorbers to make the best possible use of the incoming light to drive water splitting and fuel production with two photons per electron. A schematic of a tandem artificial photosynthesis device is shown in figure 2 along with its light-absorbing properties. Just as in natural photosynthesis, artificial photosynthesis occurs in four steps: light harvesting, charge separation, water oxidation and fuel production.

### 3.1. Light harvesting and charge separation

Natural photosynthesis has an efficient strategy for achieving light harvesting and charge separation: antennas containing many chlorophyll molecules absorb sunlight and pass the excitation energy among themselves and then through to the reaction centre. Also, secondary absorbers such as carotenes absorb light in regions of the spectrum where chlorophyll does not absorb well. In this way, the absorption cross section of the reaction centre is increased.

For artificial photosynthesis, strategies similar to that found in nature can be employed, where multiple absorbers that exhibit complementary absorption profiles and are capable of efficient excitation-energy transfer are used as light harvesters and the excitation energy is transferred to an artificial reaction centre [13–15]. For the tandem device described above, light is absorbed on the fuel production side by a photosensitizer that, when excited, has sufficient reduction potential to inject an electron into the fuel production catalyst. On the water oxidation side, the optically excited photosensitizer has sufficient reduction potential to inject an electron into the fuel reduction side and fill the hole there, and subsequently has sufficient oxidation potential to oxidize the water oxidizing catalyst.

The sensitizers may be made from organic molecules. The simplest form of this is a donor–acceptor diad [16], but more complex structures are commonly used to prevent charge recombination [17]. Alternatively, semiconductors, as in solar cells, may be used. These materials can efficiently absorb sunlight and separate charges and are stable with extended exposure to sunlight. Their disadvantage is their limited flexibility, which means that various techniques of doping, nanostructuring and coating are needed to give them the desired properties. Finally, molecular light harvesters can be used in combination with semiconductor charge separators, in a concept similar to that used in dye-sensitized solar cells [18,19].

### 3.2. Water oxidation

Recent advances in the determination of the structure of the photosystem II protein complex [20] shows the oxygen-evolving centre found in oxygenic photosynthesis has a cluster of four manganese atoms and a calcium atom surrounded by protein. This enzyme stores oxidizing potential in a series of one-electron steps and then splits water into oxygen, electrons and protons. It performs this process at low overpotential and at a very rapid rate [21,22]. As water oxidation involves multi-electron chemistry, water-splitting catalysts invariably include one or more transition metals in their structures. Some success has been achieved with water oxidation catalysts based on expensive metals such as iridium [23–25] and ruthenium [26,27]. However, these are not practical for very large-scale use. Natural photosynthesis demonstrates that catalysts based on abundant, inexpensive elements are possible. Such catalysts are more desirable. The development of artificial water oxidation catalysts that employ abundant elements is a very active research field, but an inexpensive, robust and efficient catalyst has yet to be discovered. There have been promising results with catalysts based on cobalt [28–31] and other cheap transition metals [32–35], but their efficiency and durability need to be further improved before they are suitable for larger scale use.

### 3.3. Fuel production

Different solar fuels can be envisaged as products of artificial photosynthesis. There are two main types of fuels: hydrogen and carbon-based fuels.

#### 3.3.1. Hydrogen

Hydrogen is a natural choice of fuel when water is the raw material. To make hydrogen, the protons that result from the splitting of water need to be reduced to produce molecular hydrogen:
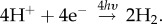


The easiest way of doing this is on the surface of a noble metal such as platinum. But this approach is too expensive to commercialize on a large scale. Hydrogenase enzymes efficiently catalyse the reversible reduction of protons and have iron and nickel centres. There has been some success in the mimicking of these catalytic centres [36,37], but the mimics mostly do not show the very high catalytic rates of the natural enzymes. More recent research suggests that modifying the second coordination sphere may improve their performance [38–40]. When combined with the water-splitting reaction in a tandem configuration, four photons are used to make one molecule of hydrogen.

Hydrogen has been identified as an attractive zero-carbon energy carrier that could play a key role in future renewable energy technology. However, hydrogen is a gas, and highly explosive when it mixes with oxygen from the air. Although large-scale hydrogen infrastructures for business to business have been operational in The Netherlands and other locations in the world with good safety record, much of our current fuel infrastructure is set up for liquid fuels and there are obstacles in the way of home refuelling relating to safety and regulation. A global project on artificial photosynthesis can play an important role in overcoming these hurdles well before hydrogen is used worldwide.

#### 3.3.2. Carbon-based fuels

Nature makes carbon-based fuels. These are complex carbohydrates with carbon–carbon bonds. However, combining protons with carbon dioxide out of the air to produce carbon-based fuels is much more challenging than producing hydrogen. This process involves difficult multi-electron chemistry, even for simple energy carriers such as methane, methanol and syngas (a mixture of carbon monoxide and hydrogen) (table 1). Artificial molecular catalysts are being developed for the photosynthesis of carbon-based fuel [41,42], and semi-synthetic systems based on enzymes from microorganisms [43] are being investigated. H_2_ and CO can be used as precursors for other fuels like methane, alcohols and Fischer–Tropsch liquids that may be incorporated into our current energy infrastructure.

**Table 1.** Two photons are needed to transfer one electron in an (artificial) photosynthetic reaction. Assuming 40 moles of photons per metre squared per day, the second and third columns show how many tons of CO_2_ can be converted per km^2^ per day and how many tons of carbon-based product can be produced per km^2^ per day, respectively. The top row shows hydrogen production as a reference.

reaction	tons of CO_2_ converted per km^2^ per day	tons of product produced per km^2^ per day
2H^+^ + 2e^−^ → H_2_	0	20
CO_2_ + 2H^+^ + 2e^−^ → HCOOH	440	460
CO_2_ + 2H^+^ + 2e^−^ → CO + H_2_O	440	280
CO_2_ + 4H^+^ + 4e^−^ → HCHO + H_2_O	220	150
CO_2_ + 6H^+^ + 6e^−^ → CH_3_OH + H_2_O	147	107
CO_2_ + 8H^+^ + 8e^−^ → CH_4_ + 2H_2_O	110	40

A further complication with carbon-based fuels is that the atmospheric concentration of CO_2_ is low. This necessitates either placing artificial photosynthetic devices near sources of CO_2_ such as coal-fired power stations or combining them with technologies that concentrate CO_2_ (see §7.4).

## 4. Challenges in artificial photosynthesis

If artificial photosynthesis is to become part of our sustainable energy mix, the systems must be efficient, durable and cost effective. The challenge is to fulfil all of these criteria. A number of systems that meet two of the three requirements have been successfully developed, but until now, accomplishing all three of them has remained elusive. Furthermore, the chemical processes that need to be performed and their associated losses all need to be fitted within the ‘energy budget’ that we get from the absorbed sunlight. To achieve this, there are many tricks we can learn from nature. Current research pushes up against the limits of physics, chemistry, nanotechnology, thermodynamics, quantum chemistry and engineering.

### 4.1. Efficient

A tandem system with cut-off wavelengths of 700 and 1100 nm makes optimal use of incoming photons and drives water splitting and fuel production with two photons per electron. The challenge then remains to optimize the yield of the chemical reactions by avoiding recombination of the charges generated by the photons after they have been absorbed. Natural photosynthetic reaction centres can achieve internal quantum efficiencies in excess of 90% in low light levels. The higher the quantum efficiency, the less material and surface area is needed. The latest generation of solar cells achieves high internal quantum efficiency, but in this respect, there is still a lot of room for improvement in artificial photosynthesis. At the moment, there are only a few very expensive systems that work with a quantum efficiency of more than 25%, corresponding to an energy efficiency of more than 10% [44].

### 4.2. Durable

Any artificial photosynthesis system will need to be durable so that it can convert a lot of energy in its lifetime relative to the energy required to install it. This is a significant challenge because many materials degrade quickly when exposed to sunlight or corrode when exposed to oxygen or water. There are two possible approaches to this issue. One is to make the system out of very robust materials, like the coated semiconductors that are used in photovoltaic cells. The other approach is to make a system that can repair itself when it is damaged. Nature takes this latter approach (photosystem II in plants is replaced approximately every half hour in high light [5]), and there have been a few experimental systems that mimic this. This self-repairing approach has the advantage that the system can then potentially also be self-assembling, making the system simpler and cheaper to build.

### 4.3. Cost effective

Any artificial photosynthesis system must be cost effective to be commercially viable. Artificial photosynthesis must compete with other technologies for the production of hydrogen (and other fuels). This means that as little material as possible should be used in its construction and rare and expensive materials must be avoided.

A recent report for the Fuel Cells and Hydrogen Joint Undertaking gave expected costs for the production of hydrogen of 2.60–3.30 euros per kilogram and 4.40 euros per kilogram at the pump for large-scale (400 kg d^−1^) production of renewable hydrogen with electrolysis technologies [45]. This is in line with hydrogen costs of $3.10–$3.70 per kilogram projected by NREL [46] and can serve as a benchmark for a global artificial photosynthesis initiative. To make artificial photosynthesis as cheap as possible, these systems may ultimately be made locally from plastics using three-dimensional printing technologies.

### 4.4. The energy budget

The cut-off wavelength at which the energy is absorbed determines the potential at which electrons are produced. For 700 nm, this is 1.8 V, and for 1100 nm it is 1.1 V. If we make a tandem artificial photosynthetic device with cut-off wavelengths of 700 and 1100 nm, we have a total of 2.9 V for energy storage and to cover entropic and resistance losses. Assuming our device produces hydrogen, the following functions need to be performed by the device within the 2.9 V budget.

— Split water. The standard redox potential for water splitting is 1.23 V, but it will not proceed at this potential. An extra 0.25 V is needed for thermodynamic reasons giving a total of 1.48 V, the thermoneutral potential.
— An extra 0.5 V needs to be added to stop light reactions running backwards.
— The catalysts have overpotentials associated with them. Water splitting is a complex reaction and the overpotential is about 0.4 V. For hydrogen production, the overpotential is about 0.2 V.
— Protons need to be transported, which takes about 0.2 V.
— Finally, the hydrogen will need to be compressed for storage. This takes at least about 0.2 V (depending on the pressure required).

These are estimates based on computer models of systems for artificial photosynthesis [47]. The sum of all these potentials is 2.98 V, and from this it can be seen that in practice it is very difficult to perform this reaction with 2.9 V. Of this 2.9 V, only 1.23 V are actually used to store energy in the chemical bonds of hydrogen, which puts an upper theoretical limit on the artificial photosynthesis energy efficiency by quantitative conversion of photons of 42% for hydrogen. As a practical limit 31%, is nowadays considered realistic, as losses from, for example, reflection at the surface and a limited filling factor will have to be taken into account [47]. For carbon-based fuels, the situation is worse, because approximately an extra 0.5 V in overpotential is needed for a CO_2_ reducing catalyst. Furthermore, CO_2_ will need to be concentrated from the atmosphere.

For a global artificial photosynthesis project, it is important to realize that according to the second law of thermodynamics, a round trip from electric charge in the tandem to hydrogen and back to electricity will always lead to significantly less energy than when electricity is used directly, because of the mixing entropy that has to be generated in a chemical conversion process to obtain the highest forward yield against wasteful back reactions, given by the equation [48]4.1
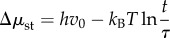
where 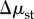 is the chemical potential of energy storage. The first term in equation (4.1) is the energy of the highest occupied molecular orbital–lowest unoccupied molecular orbital gap for light absorption and the second term is the mixing entropy. *t* is the lifetime of the storage reservoir and *τ* is the excited state lifetime of the absorber. The longer the energy needs to be stored, the more mixing entropy needs to be generated in the chemical network to prevent light reactions running backwards. The rate limiting step in the catalysis of water oxidation occurs on a time scale of milliseconds or longer. Equation (4.1) tells us that storage of an electron on this time scale requires a mixing entropy of at least 0.4 eV, in line with the number given above for preventing light reactions from running backwards. For energy storage on longer time scales, more mixing entropy is needed. For instance, at the thermoneutral potential of 1.48 V, an additional 0.25 eV of energy is available to generate the mixing entropy for the irreversible release of oxygen gas in the environment.

One of the key aspects of artificial photosynthesis is that it directly converts sunlight into fuel without any biological, chemical or physical intermediates. Additional conversion steps inevitably lead to further thermodynamic losses and thus further reduce the ‘well to wheel’ efficiency.

For natural photosynthesis, the energy budget is 3.6 V, as both stages operate with the same energy gaps corresponding to 1.8 V each. In addition, nature has a very clever trick to fit the thermodynamic requirements into the available energy budget. Rather than performing a sequence of thermodynamic conversions that each generate mixing entropy along the way according to equation (4.1), photosynthetic conversion minimizes entropy production by exploiting synergy between the steps. One overpotential is used for—sequentially—preventing recombination, the kinetic stabilization of intermediates in catalysis and finally generating targeted heat for the release of oxygen [49]. The protein matrix in which the active components sit achieves this feat. We believe that the same trick needs to be performed in an artificial system based on an artificial ‘responsive matrix’ for optimal performance [50]. A critical challenge in artificial photosynthesis research is thus to learn from nature how to engineer materials in such a way that entropy production is delayed by exploiting as much as possible isoenergetic transitions between nearly degenerate electronic states that can be made unidirectional by balancing quasi-quantum coherence with decoherence [51].

### 4.5. The responsive matrix

The working principles of natural photosynthesis act as a blueprint for the production of artificial photosynthesis. One of the most intriguing aspects of nature's success is the use of ‘responsive matrices': the various components (antennas, charge separators, multi-electron catalysts for water splitting and fuel production) in photosynthesis are pre-programmed by their protein environment for optimal operation in their given function, and the entire photosynthetic process is driven forward with minimal losses.

Chlorophyll takes on a number of different roles in plant photosynthesis: it acts as a light harvester, it dissipates excess excitation energy under high light levels and acts as a charge separator. There are negligible structural differences between the chlorophylls performing these different functions. Instead, these differences are almost certainly driven by the matrix altering the local dielectric constant around the pigment, which in turn alters the electronic properties of the chlorophyll [52,53].

The matrix plays a key role in direct injection of electric charge from the reaction centre to the oxygen-evolving centre, stabilization of the catalytic centre and control of reaction rates such that charge separation, catalysis and heat release all happen in a coordinated manner. Without direct injection of charge from the reaction centre to the oxygen-evolving centre, the electric charges would diffuse, after which they must be concentrated leading to additional losses. Proper control of the catalytic centre enables the chemical bond between oxygen atoms to be formed immediately before releasing the oxygen gas [54]. In this way, any heat that has been generated in the process is used optimally for both oxygen formation and release. If the O = O bond is formed too early, then more heat needs to be generated to liberate the oxygen, thus reducing the efficiency of the overall process.

Embedding the photosynthetic components into a responsive matrix has enabled nature to construct a self-assembling, self-repairing system from a limited set of materials that operates with a high efficiency. How nature performs this feat is not yet well understood, but much progress has been made in the understanding of how this matrix works with the help of ultrafast spectroscopy, high-resolution solid-state NMR and quantum-mechanical modelling [55–57]. In recent years, there has been a growing body of evidence to suggest that coupling with vibrational modes plays a key role in the functioning of the responsive matrix [58]. Proteins are materials with handedness that sustain a specific long-living collective vibration. They react to sudden antibonding character invoked by excitation with light or extraction of charge with a two-step response: they undergo a concerted motion that gradually decreases the energy separation between the chemical reactant and the chemical product states; when this separation matches the energy of a collective vibration along a different normal coordinate, the time scales of electronic and nuclear motion converge and resonance occurs that opens up a channel through a catalytic barrier for lossless quantum coherent conversion of the reactant into the product.

### 4.6. Vibronic coupling and the responsive matrix

In much of chemistry, the quantum-mechanical state is not influenced by nuclear motion. This arises because the nuclei and the electrons have very different masses and therefore very different relative speeds: when the nuclei move, the electrons are assumed to adjust instantaneously. The system remains in the same electronic state. A simple example of a reaction that occurs under this regime is an electron transfer reaction where the electron is initially localized on a donor and then ends up on an acceptor as a result of the electrons adapting to the motions of the nuclei. Such a reaction is known as adiabatic.

In contrast to adiabatic systems, non-adiabatic systems involve more than one electronic state. Examples of this are photoactive systems where energy or electron transfer is induced by the absorption of a photon, or catalysts where catalytic conversion is initiated by the injection of electronic charge. Recently, the quantum dynamics trajectories for a minimal electron transfer system were theoretically explored in depth and it was found that a slow and deterministic electron transfer process can be driven by a rapid molecular vibration [59]. This concept can be expanded to also describe exciton transfer, charge transfer or catalysis. In appendix A, it is explained how non-adiabatic coupling between two electronic levels interacting with a single nuclear mode opens up a fast reaction channel through an ‘avoided crossing’ of energy levels. The vibration induces a complicated spiral electronic trajectory in the responsive matrix that slowly converts the photon excitation into a charge separated state, in many ways similar to the process of nutation in NMR [60]. When viewed from the interaction frame of the component of the nuclear rotational motion that couples to the electron dynamics (similar to the rotating frame in NMR), the rapid rotation appears quenched, the two levels are separated by the non-adiabatic or vibronic coupling and the nutation reduces to a simple rotation of the two electronic states by quasi-quantum coherence. In photosynthesis, the near unity quantum yield of photon-to-charge conversion is thought to arise from similar coupled nuclear and electronic motions in a responsive matrix [61–64]. In these systems, the matrix couples specific vibrations to the photoexcited pigment to remove the barrier to charge separation, much like the promoting vibrations observed in enzyme catalysis [65].

**Figure 3.**
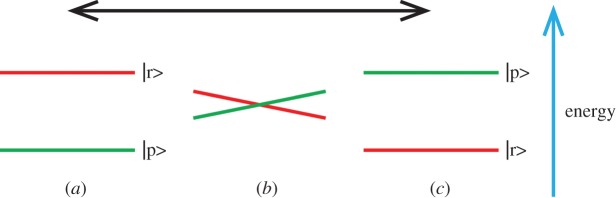
The principle of the responsive matrix for the conversion of a quantum chemical reactant state |r> into a product state |p> by means of coupling to a molecular vibration. In (*a*), at the start of the oscillation (indicated by the double-headed arrow at the top of the figure), the states are energetically well separated and the system is in state |r>. Both |r> and |p> are pure quantum states. The molecular vibration brings the two states periodically together (*b*) so that they overlap and become quantum mechanically unstable. The state |p> is then in a coherent superposition with the state |r>. After half a period of the vibration (*c*), the energy levels of |r> and |p> are exchanged. When the nuclear dynamics is synchronous with the electron dynamics, resonant quantum conversion between the two states is enabled. After several periods of oscillation *abcba*, a chemically pure reactant state |r> is converted to a chemically pure product state |p>. (Online version in colour.)

To complement the treatment for the expert in appendix A, where the quantum propagator *ρ*(*t*) for the process is derived, a simple schematic diagram of the electronic states is shown in figure 3. States |r> and |p> are two pure states, for the chemical reactant and product, but they are not eigenstates when |r> and |p> are close in space and close in energy. Off-diagonal terms in the Hamiltonian then lead to (transient) avoided crossings of the |r> and |p> levels, a phenomenon that is well known in photochemistry [66]. |r> and |p> are the only states that can interact against a background manifold of states that are energetically well separated and are coupled to a normal mode *ω*_n_. The energy difference between |r> and |p> starts to fluctuate, driven by the coupling to *ω*_n_. When these fluctuations are faster than the dephasing time of the quantum system, then |r> and |p> will be in a coherent superposition (figure 3*b*), allowing conversion from state |r> to state |p> (figure 3*c*) [67,68]. This conversion process is mathematically described by a propagator in Liouville space4.2
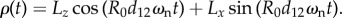

Dynamic symmetry breaking is crucial for such a vibrationally coupled electron transfer process because the molecular oscillation has to couple to coherent dynamics of the electronic quantum states. Without symmetry breaking, crossing of the electronic levels is forbidden.

This is an example of the so-called ‘quantum Goldilocks effect’ [51,69]. This effect is named after the English fairy tale about a girl who tries out various items in the house of the three bears in search of items that are ‘just right’ (porridge that is not too hot or too cold, beds that are not too hard or too soft, etc.). In the case here, the vibration and level of decoherence have to be ‘just right’. If there is only noise, the system will never settle into a well-defined state. If there is too little noise, then back transfer from |p> to |r> can also occur coherently, which is obviously less efficient. In this way, nature has evolved a mechanism to optimize and control reactions in photosynthesis [51,58].

This mechanism enables the coherent conversion of one electronic state into another by the exchange of angular momentum without the need for a redox ladder or other kind of energy level difference. In this way, photocatalysis can occur with optimal efficiency. We believe that this principle is at the heart of the responsive matrix and will also be crucial to obtain high efficiencies in artificial photosynthesis. The quasi-quantum coherence and its associated dynamics is robust and suitable for application in artificial photosynthesis, as quantum dynamical networks select the appropriate *ω*_n_ for fast dynamics from the available vibrations in the matrix. The matrix can be structurally programmed to provide a long-living *ω*_n_ that can be used for a fruitful propagation of |r> into |p> [56].

Thus, the non-adiabatic regime is a very interesting one to pursue. Research in this direction would benefit from a global consortium as it is highly interdisciplinary: there is still more to be learnt from nature, the basic physics and chemistry needs to be more thoroughly understood and the route to application must be sought (see §7.1). There are a wide range of materials for artificial photosynthesis (see §5) under investigation in the BioSolar Cells consortium (see §6) and other research consortia around the world. These provide a wide array of substances for examination as potential responsive matrices. Then, any future device should be designed such that all processes in the total solar-to-fuel conversion occur with minimum loss and optimal use is made of the responsive matrix. Finally, any economic, environmental and social constraints should be taken into consideration. All these steps would be well supported by the collaborations and resources of a global consortium.

## 5. Approaches to artificial photosynthesis

There are a number of different approaches to artificial photosynthesis that can be employed in a global initiative. They share the four basic steps of light harvesting, charge separation, water splitting and fuel production. The scientific problems encountered in all cases are similar. Here, we have divided up the different approaches according to the types of materials used: organic, inorganic, hybrid and semi-synthetic.

### 5.1. Organic systems

Molecular artificial synthesis components are often developed biomimetically [13,70,71]. Studies of energy conversion and storage in efficient natural enzymes provide inspiration for the development of the complex chemistry by mimicking enzymatic catalytic functions. The four-electron oxidation of water is a main research problem and a bottleneck for successful development of artificial photosynthesis [72]. Entirely molecular systems are difficult to develop, but they offer the advantage of enabling a modular approach. Individual components for light harvesting, charge separation, water oxidation and fuel production can be made and investigated separately to maximize performance before being integrated into an appropriate architecture. Molecules have a well-defined structure that can be deliberately modified to improve a given property. Molecular systems are also readily amenable to study with analytical techniques that provide structural and kinetic information. Processes can therefore be followed and understood at a very detailed level. This molecular assembly approach is elegant, but in the absence of a viable spontaneous assembly strategy, the large amount of synthesis involved renders it impractical [73]. Also, most molecules tend to degrade quickly under extended exposure to sunlight. Should scientists obtain a greater understanding of ‘responsive matrices' that would enable self-assembly and self-repair, then these molecular systems may become the champions.

### 5.2. Inorganic systems

We know from solar cells that semiconductor materials can absorb sunlight and then separate charges. Also these materials are generally robust under extended exposure to sunlight. Semiconductors therefore seem to be natural candidates for artificial photosynthesis. Those with appropriate electronic properties can provide sufficient electrochemical potential to drive water oxidation or fuel production on their surfaces. However, many semiconductor materials that have the right electronic properties for water splitting only absorb UV light, which is only a small portion of the incoming photons. Furthermore, catalysis on the semiconductor surface is not very efficient. Although appealing for its simplicity, having one semiconductor perform all tasks of absorption, charge separation and catalysis is asking a lot from one material [73]. To date, no material with acceptable performance has been found. To address this significant challenge, novel concepts and methods afforded by nanotechnology are being applied to design innovative composite nanostructures in which each component performs specialized functions [74–76].

### 5.3. Organic–inorganic hybrids

An appealing solution is to combine the best properties of organic and inorganic materials. Here, light absorption may be done either by a semiconductor or by a dye molecule on a semiconductor surface; charges are then separated within the semiconductor and transported to optimized molecular catalysts tethered to the semiconductor surface. This is a very promising approach, and, to date, a number of experimental devices have been constructed this way [18,77–80]. However, many are still either too expensive or too inefficient to warrant scaling up to a commercial level.

### 5.4. Semi-synthetic systems

An interesting new approach concerns systems that are a hybrid of biological and man-made components. For instance, a biological photosynthetic component that harvests solar energy and splits water can be purified and tethered to an appropriate scaffold. The photosynthetic enzyme is then linked to a hydrogen-producing enzyme (a hydrogenase) or a catalyst for fuel production [81–83]. Alternatively, chlorophylls from biological sources can be chemically modified and assembled to form semi-artificial modules [84–87]. The advantage of this approach is that we know that the biological components work very well (nature has been successfully performing photosynthesis for about 3 billion years). However, this method is in its infancy, so it remains to be seen if these biological components can be made sufficiently robust by chemical modifications outside of their native environment, or if they can be extracted and modified in a commercially viable fashion. Even if such constructions never yield commercially viable devices, the science involved in studying them promises to teach us a lot about nature's approach to photosynthesis.

## 6. Research on artificial photosynthesis in The Netherlands

The major research initiative on artificial photosynthesis in The Netherlands is the BioSolar Cells programme. This public–private partnership was established in 2010. The programme is funded by The Netherlands Organisation for Scientific Research, the Dutch Ministry of Economic Affairs, Agriculture and Innovation and the companies and universities that make up the consortium. The BioSolar Cells programme has three themes: artificial photosynthesis, photosynthesis in cellular systems and photosynthesis in plants. These three research themes are underpinned by a fourth theme: education and societal debate, where educational modules are developed, and the societal consequences of new solar-to-fuel conversion technologies are debated in public.

The artificial photosynthesis section of BioSolar Cells aims to produce at least two artificial leaves that use sunlight to split water into oxygen and hydrogen. To explore all avenues to overcoming the challenges described in §4, all methods described in §5 are being researched. There is also significant work on theory and analysis to deepen our understanding of the artificial systems developed within the programme and their natural counterparts.

A number of molecular catalysts for both water oxidation [23,25] and proton reduction [88] have been developed. These are now being improved to increase turnover frequency and robustness. Also, a number of light-harvesting molecules have been made [89,90]. A key strength of molecular systems is that the catalyst that drives the reaction directly quenches the excited state generated by the photon. In principle, such an approach enables the absorption of the photosensitizer to be very well matched to the potentials of the reactions, leading to improved efficiency. Molecular systems that perform light-driven catalysis have been developed [88,91]. As well as studying these light-driven reactions in solution, immobilization strategies are also being investigated to improve the usability of these systems.

**Figure 4.**
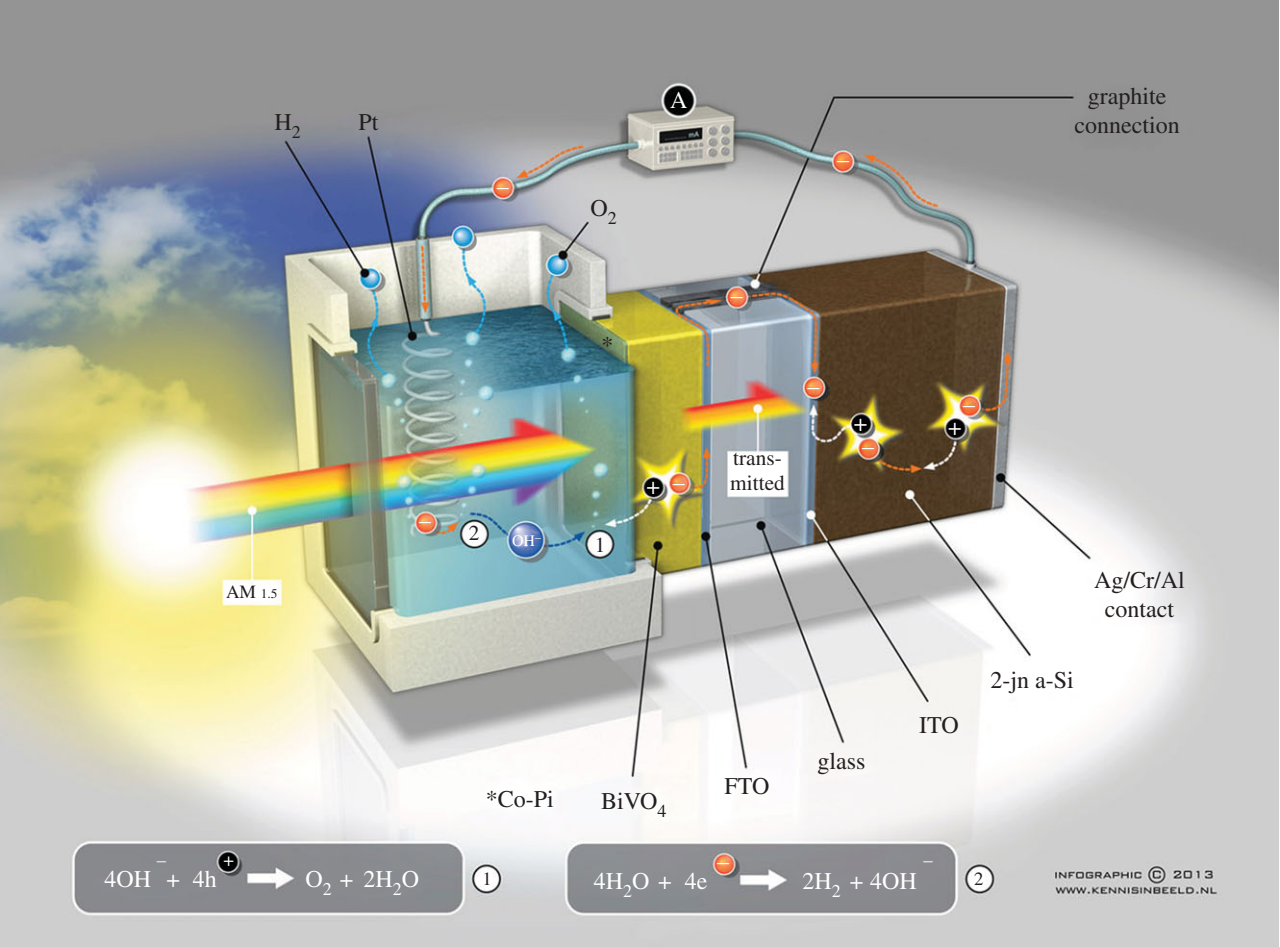
An ‘infographic’ representation of the water splitting device made of a combination of gradient-doped W : BiVO_4_ and a-Si solar cell as described in [74]. (Online version in colour.)

Another part of the programme works on the nanofabrication of semiconductors to fine tune their properties for use in artificial photosynthesis. In such a device, the semiconductor performs light harvesting and charge separation. Water oxidation takes place either directly on the semiconductor surface or with the help of a catalytic coating on the semiconductor. Proton reduction is often performed on a noble metal surface such as platinum. An example of such a device produced within the BioSolar Cells programme is a bismuth vanadate–silicon triple junction photoelectrode [74]. A schematic of this device is shown in figure 4. Also within the BioSolar Cells programme, InP nanostructured devices are being worked on [92]. In a related part of the programme, multi-junction plastic solar cells have been made that, in combination with platinum, give approximately 4% solar to hydrogen efficiency [93].

Many of the molecular catalysts for proton reduction and water oxidation that have been prepared within the consortium have been functionalized with groups that enable immobilization onto various surfaces including semiconductors. This enables the construction of organic–inorganic hybrid devices. However, not all of the catalysts have proved optimal for functioning on semiconductor surfaces, so further improvement is necessary.

Also, there is a semi-synthetic system being worked on to make carbon-based fuel. Here, the biological metabolic reaction pathway of three dehydrogenases, formate dehydrogenase, formaldehyde dehydrogenase and alcohol dehydrogenase, is reversed, enabling the transformation of CO_2_ into methanol by a cascade reaction. This cascade reaction consumes NADH. When this reaction can be combined with the light-driven regeneration of NADH, then an artificial photosynthesis device that produces a carbon-based fuel is formed.

To verify the experimental data and understand the functioning of the various systems, spectroscopic and theoretical research plays an important role. The molecules and materials are studied to obtain information about their structure and function as individual components or in combination with other parts. Computer calculations give information about how the catalytic reactions take place through coupling between molecular vibrations and the movements of electrons, and what energies are required for this, so that components can be modified for better functioning.

The underpinning educational and societal debate section develops educational modules and explores the societal context for new BioSolar Cells technologies. In the educational section, the aim is to develop high-quality educational programmes on the subject of (natural and artificial) photosynthesis to qualify and inspire future researchers, policy makers and industrialists. Furthermore, the interest and motivation of good students can be harnessed to acquire knowledge for the programme and help steer its direction. The educational material developed so far consists of modules for high school students, through to courses for bachelor and masters students and short schools for PhD students from within the consortium and from related programmes. Education at the high school level is a form of outreach, bachelors and masters education helps to train and equip future generations, while PhD student schools provide a platform for collaboration and help to develop additional vision for the programme. The societal debate is important for the implementation of the science. The results of the research can only be implemented if they fit into a socially, economically and ecologically sustainable context. This context is researched in BioSolar Cells and the results fed back into the scientific programme. To date, a philosophy of energy is under development to shed light on what the key issues are in the energy transition [94]; life cycle analysis models for BioSolar Cells technologies are being constructed [95]; the role of public engagement is being studied; and a field of interpretation for BioSolar Cells is being explored through the means of bioart.

## 7. Artificial photosynthesis in society

### 7.1. A global artificial photosynthesis consortium

Research into artificial photosynthesis is very interdisciplinary. The challenges are physical, chemical, biological and technical. Knowledge from chemical sub-disciplines such as photochemistry, electrochemistry, materials chemistry, catalysis, organic chemistry and biochemistry is of great importance. From this point of view, mechanisms that promote collaborations between scientists are very valuable. The BioSolar Cells programme, described above, can be seen as a microcosm of a global artificial photosynthesis consortium [96]. Here, scientists who specialize in various aspects of artificial photosynthesis (light harvesting, charge separation and catalysis, as well as forms of analysis and device building) learn from each other and work towards a common goal. Such a consortium should provide mechanisms for scientists to meet regularly and exchange ideas and enable interactions with scientists working on natural photosynthesis as it is the blueprint for artificial photosynthesis. Furthermore, there should be room to bridge from basic science to engineering problems to questions of usability and interactions with end users. Interactions with society are important for determining the limits on social acceptance of new technologies and investigating viable structures for the implementation of these technologies. In the sections that follow, we will look at some of these societal aspects.

### 7.2. New business models for the distribution of energy

The route from laboratory prototype systems to commercial technologies is still long for artificial photosynthesis. However, renewable energy industries can develop very rapidly once commercially viable—the growth seen in the silicon photovoltaics industry is a prime case. People are often proud of having solar panels on their roof and feel they make a useful contribution to society when they can produce their own electricity. Furthermore, when an individual has a large area of solar panels, they return excess electricity to the grid and are financially compensated for this. The consumer sells energy and becomes a producing consumer: a so-called prosumer. But, as has been previously pointed out, the electricity that solar panels make is not easy to store, which limits the possibilities for the consumers to really become prosumers. Artificial photosynthesis does not suffer this problem; these devices produce fuel, making energy available to the owner day and night. Products will be produced when the Sun shines, even if there is no demand. Under these circumstances, the owner is in a position to sell fuel. For instance, in The Netherlands, the existing natural gas grid has a storage capacity of approximately 500 TWh and can accommodate hydrogen up to approximately 10% of the total capacity. This makes it possible for early adaptors to inject their hydrogen production into the gas grid. With such a model, the enterprising middle class citizen has the opportunity to participate in the energy economy. It is a logical extension to the current practice with solar cells producing electricity. In this way, everyone can become a part-time fuel producer. The financial returns from energy production will probably not be large, but it is the sense of pride and well-being that people get from producing their own energy that will initially drive the uptake of this technology. Furthermore, the people who need fuel can produce it at the location where it is needed. Thus, fuel transport costs can be reduced and resource-recycling loops can be closed locally [97]. Such a model radically changes our energy ethic [98].

In the longer term, individuals will need to join forces to promote their interests and organize the distribution of their excess products [99]. This may lead to a change in the roles of utility companies as not so much monopolizers of energy production, but as energy distributers, as their knowledge of energy distribution networks will be invaluable here.

Another potential advantage of the large-scale implementation of artificial photosynthesis arises from the relatively even spread of solar energy over the planet (in contrast to fossil fuels). Should artificial photosynthesis be deployed the world over, then people in poor countries will have access to the necessary energy resources to improve their standard of living.

### 7.3. Renewable fuels for transport

Solar hydrogen can be used to power hydrogen vehicles. In April 2013, Hyundai introduced the ix35, a car with a hydrogen fuel cell. Several other car manufacturers, such as BMW (Hydrogen 7) and Honda (FCX Clarity), have released limited editions of cars with similar technology or with engines that burn hydrogen. General Motors, Daimler Ford, Nissan and Toyota are still working to make affordable fuel cell cars.

Fuel-cell-grade hydrogen is currently produced by steam reforming, which is energy intensive and produces greenhouse gases. One of the challenges for hydrogen transport, therefore, is to find alternative ways to produce hydrogen. On average, there are 10–120 moles of photons per metre squared per day incident on the surface of the Earth. Can we realistically use these photons to produce hydrogen to drive a car? It was shown that we would need 4 moles of photons to make 1 mole of hydrogen. Thus, 2.5–30 moles of hydrogen can be produced per square metre of artificial photosynthesis per day. One mole of hydrogen is equivalent to 2 g of hydrogen; which means that 5–60 g of hydrogen is produced per metre squared per day. A car like the Honda Clarity would need about 500 g of hydrogen per day. This means that in sunnier locations about 10–20 m^2^ of artificial photosynthesis (about the same area as the garage that the car is kept in) is sufficient for the owner of the car to produce enough hydrogen to power their own car once the technology is fully developed to operate at high photon to fuel quantum yield.

Progress is being made with other challenges such as setting up a distribution network for hydrogen, storing hydrogen and improving fuel cell technologies [100]. If hydrogen were produced using sunlight, this would be a major step forward in enabling its use as a transport fuel on a large scale. Artificial photosynthesis, being intrinsically a decentralized technology, will also not deliver energy at the high concentration that is needed for our industries and for transportation with trucks, ships and planes without accompanying measures, such as a grid to collect the fuel. Such infrastructure could be considered in the context of a global artificial photosynthesis initiative.

### 7.4. Carbon use and recycling

Increasing emissions of carbon dioxide from the combustion of fossil fuels have been identified as the dominating factor behind global warming. Various proposals have therefore been made for the capture and long-term storage of carbon dioxide. But rather than long-time storage of carbon dioxide captured from fossil-fuel-burning power stations—a major challenge in itself—an attractive prospect is to use it to produce fuels and feedstock.

Sunlight could provide the energy needed to produce fuels from CO_2_. Carbon capture technology could provide a source of CO_2_ which, when combined with water and solar energy, could produce carbon-based fuels such as carbon monoxide, formic acid or methanol. Recent analyses performed by BioSolar Cells indicate that producing hydrocarbons from CO_2_ for aeroplanes and trucks using existing technologies produces more CO_2_ and uses more energy than fuel production from existing fossil sources. An improvement in life cycle CO_2_ emissions is expected when solar energy and atmospheric CO_2_ are used, as in artificial photosynthesis. Also for levelling supply with demand in smart grids the decentralized production of hydrocarbons by artificial photosynthesis is one of the technologies that merits further exploration [95].

In a western European country such as The Netherlands, on average there fall 40 mol photons per square metre per day. (Artificial) photosynthesis converts CO_2_ with two photons per electron. Table 1 shows how many tons of CO_2_ can potentially be converted per km^2^ per day for various CO_2_ conversion reactions. Formic acid, syngas, formaldehyde, methanol and methane are listed as possible carbon-based fuels.

## 8. Conclusion

Researchers worldwide are working on different ways to make hydrogen and carbon-based fuels using nothing but water, carbon dioxide and sunlight as raw materials. These fuels provide the possibility to store and transport solar energy, giving us access to this energy at any time anywhere in the world. The fundamental barrier to achieving this is creating a device that is simultaneously cheap, robust and efficient. We believe that this can be solved by unravelling secrets from nature such as how to deploy a responsive matrix and the role of vibronic coupling. In research programmes around the world, large efforts are being made to solve these and other problems and bring viable prototype devices into existence. The next step is artificial photosynthesis at pilot scale, approximately 100 m^2^. A global consortium on artificial photosynthesis will give these efforts momentum, while at the same time raise the visibility of this potentially game-changing technology.

Technology for the conversion of sunlight to fuel can contribute to the closing of loops on both the large and the small scale. It remains to be seen exactly how these technologies will be applied and what the social and economic consequences are. Science has made this scenario a possibility, but to make it a reality the insights of research must be supported by the incentives of economics and the political will of countries across the globe. The prospects are exciting and we can look forward to where a global project on artificial photosynthesis may take us.

## Appendix A. Quantum coherent conversion with responsive matrices

Here, we present a first principles description of how activation of a nuclear mode can produce convergence of time scales of quantum electron dynamics and classical nuclear dynamics by symmetry breaking, and how this opens a reaction channel through an avoided crossing between two electronic states. The result is an effective pathway for a slow and lossless conversion of reactant to product by quasi-quantum coherent evolution, for which a quantum propagator is derived that can be associated with rotational dynamics promoted by a molecular environment with handedness to break the symmetry. The strength of the nuclear mode determines the reaction rate, which can be controlled with a chemically engineered responsive matrix structure.

The electronic and nuclear states of the responsive matrix components comprising a photochemical conversion and concentration chain for natural or artificial photosynthesis can be described by linear combinations of electron–nuclear (vibronic) wave functions

A 1

These wave functions are the solutions of the time-independent Schrödinger equation

A 2

with *E_I_*(*R*(*t*)) the energy eigenvalues. Without simplification, the Schrödinger equation describes all the particles (electrons and nuclei in this case) simultaneously [101].

The Hamiltonian is composed of two terms

A 3

where  is the nuclear kinetic energy operator with *M_i_* the mass of the *i*th nucleus.

A 4

is the electronic Hamiltonian. Here,  is the kinetic energy of the electrons with *m* the mass of the electron and *V*_el_ (*r*, *R*(*r*, *t*)) is the potential energy that includes electron–nuclear, electron–electron and nuclear–nuclear interactions.

The dynamics of the chemical conversion is described with the time-dependent Schrödinger equation of motion

A 5

When the separation between energy levels is large compared with the kinetic energy of the nuclei, the electrons adjust instantaneously to shifts in nuclear positions owing to the very different masses (and therefore relative speeds) of the nuclei and electrons. Under this regime, known as the Born–Oppenheimer approximation, the vibrational and electronic parts are separable. The vibronic wave functions are written as product states of electronic  and nuclear  components for the *i*th electronic and *a*th nuclear state

A 6

The double index  denotes the wave functions corresponding to the *i*th electronic and *a*th nuclear state.  are the eigenstates of a pseudo time-independent Schrödinger equation for every nuclear configuration

A 7

The  form at any point in time a basis set spanning the subspace of the electronic states.

To allow for non-adiabatic couplings between electronic states, we sum over vibronic states and treat the nuclear motion as a time-dependent parameter

A 8

that is indexed by the electronic state *i* and matches the time evolution of the  in the electronic Hilbert space to assure convergence of time scales of nuclear and electronic motion. By substituting equation (A 6) into equation (A 1) and using equation (A 8), the total time-dependent wave function can be written as

A 9

The  now play the role of time-dependent coefficients that measure the contribution of the  in the total wave function.

It can be shown [101] that if equation (A 9) is expanded for an arbitrary number of electronic states and substituted into equation (A 5), a set of generalized Schrödinger equations

A 10

is obtained for the time-dependent coefficients  that describe the electron–nuclear dynamics. Here, the  are first-order non-adiabatic couplings between states *i* and *j*. The *E_j_* are the energies of the electronic system that appear after integrating out the electronic part of the wave function. Equation (A 10) is valid within a quantum–classical treatment since here we have substituted the quantum-mechanical operator for linear momentum with the classical quantity *p*.

For driving a non-adiabatic transition, we consider the system filtering a single long-living normal mode *ω*_n_ out of the many modes that are present, with displacement *R*_0_ and phase *φ*, and with its associated momentum . This leads to

A 11

Non-adiabatic transfer requires an energy difference between electronic states that is small compared with the kinetic energy of the nuclei. Hence, we reduce the system to a subspace of the Hilbert space spanned by two states *Φ*_1,2_ that can interact, while neglecting the background manifold of states that are energetically well separated. As all  are by definition eigenstates of a pseudo time-independent Schrödinger equation (equation (A 5)) in the adiabatic regime, the *Φ*_1,2_ can be conveniently chosen to represent the process that is under consideration, energy transfer, charge separation or catalysis. This leads to two time-dependent states coupled to the same long-living nuclear mode *ω*_n_:

A 12*a*

A 12*b*

Here,  are the time-dependent coefficients that describe the dynamics, just as in equation (A 9), and *Φ*_1,2_ are two orthonormal pure electronic states. When considering only two states, equation (A 11) becomes

A 13

By choosing *E*_1_ = *E* and *E*_2_ = −*E*, the energies of the electronic states are symmetrically positioned around zero, and  as the matrix describing the interaction is Hermitian. Also, we choose *φ* = 0. Using the Pauli spin matrices  and , equation (A 13) can be written as

A 14

The  transform in Hilbert space as angular momentum operators  and , and the time dependence of the electronic levels in the non-adiabatic coupling regime can be viewed analogously to precession for an *S* = 1/2 fictitious spin [102]. Hence, we arrive at a time-dependent Hamiltonian for the spinor

A 15

where *ω*_0_ formally represents a precession frequency associated with coherent dynamics of the electronic states and is related to the energy difference between the two states (*E*_2_ – *E*_1_). The convergence of time scales of electron and nuclear dynamics that enables non-adiabatic transfer to occur happens when *ω*_0_ = *ω*_n_. It is then relatively straightforward to remove the implicit time dependencies in the equation of motion and the wave functions by transforming the Schrödinger equation (equation (A 14)) to the interaction frame of the nuclear motion [61]. This can be achieved by decomposing sin(*ω*_n_*t*) into two components that counter rotate along ***L****_z_* using the transformation rule for exponential operators

A 16

whereby

A 17

The first term rotates ***L****_y_* at a frequency *ω*_n_ around the *z*-axis and leads to the convergent, Goldilocks-type electron–nuclear dynamics. The second term, which rotates ***L****_y_* at a frequency −*ω*_n_ around the same axis, has a mismatch of 2*ω*_n_ with the electronic motion. This term does not give rise to vibronic coupling and can be neglected. Model calculations [59] of coherent transfer with Ehrenfest dynamics have shown that imposing strict symmetry on a minimal model where the two relevant electronic states are an exciton state and a charge transfer state quenches the charge transfer, which can proceed smoothly when symmetry breaking is allowed. It is then tempting to conclude that the ubiquitous chirality of biological systems facilitates the necessary symmetry breaking for non-adiabatic conversions, which should be important for the design of artificial photosynthesis responsive matrix components with optimal chemical reactivity. To remove the time dependence of *H*_1_, we need to perform a transformation

A 18

Likewise,

A 19

Hence the Hamiltonian in the interaction frame of the nuclear motion

A 20

is time independent.

In this interaction frame, the wave functions are

A 21

As the  are time-dependent probabilities, and any *R*-dependence is implicitly motion in a circular orbit, they are not affected by a complex phase factor. We can use the same choice of rotation direction for the electronic oscillations as was made for the nuclear vibrations to remove the implicit time dependence from the electronic part of the basis set to recover the pure and time-independent  and  to span the Hilbert subspace, while maintaining the  to describe the dynamics. As in the interaction frame, the nuclear rotation matches the electronic rotation with *ω*_0_ − *ω*_n_ = 0, a simple time-independent non-adiabatic interaction Hamiltonian

A 22

induces coherent transfer between the states  and . The transfer is described by two coupled differential equations

A 23*a*

A 23*b*

with solutions

A 24*a*

A 24*b*

For an initial state  at *t* = 0,  and . After a time ,  and , and the reactant is fully converted into the product by semi-classical coherent evolution through a superposition wave function

A 25

In addition to using wave functions, it is instructive to move from Hilbert space to Liouville space and describe the conversion within the density operator formalism. As the states transform according to angular momentum operators in the equation of motion (equation (A 14)) and rotational symmetry breaking is essential to couple only one of the two counter rotating components of the nuclear vibration to the electron dynamics, the density operator at the start of the conversion is proportional to

A 26

by the extent *ε* of the symmetry breaking.

The equation of motion

A 27

then leads to the propagator

A 28

and after half a period the reactant is transformed into the product by the non-adiabatic coupling through a transfer of angular momentum from the nuclear system to the electronic system, corresponding with a change of the density operator from ***L***_z_ to −***L****_z_* along an isoenergetic evolution trajectory.

While spontaneous symmetry breaking  in the electron dynamics may occur because of instabilities, we anticipate that the efficiency of the quantum process will increase when electron dynamics is activated prior to the reaction by long-living vibrational coherence and the handedness of the supporting matrix. A convenient way to achieve this is by the chemical programming of induced misfits in responsive matrix components, similar to natural photosynthesis [56]. The vibrational enhancement prior to the reaction can then involve a second collective mode  for the motion along the coordinate of the cooperative twisting change of the structural environment upon activation by light absorption or charge injection. Hence, the normal modes in the ground state will not be orthogonal after activation, which should give rise to cross terms to facilitate the desired conversion. Thus, rather than using responsive matrix components based on one engineered conformational equilibrium, it is more productive to enhance the electron dynamics prior to the reaction with a conformational equilibrium and then switch over to a second conformational equilibrium to actually perform the reaction. This is how proteins do it. For instance, recent simulations of coherent charge transfer in photosynthetic reaction centres have revealed activation of the special pair by twisting motion in the ground state first and then a further rotation in the excited state coupled to rearrangement of the surrounding matrix involving proton displacements accompanying the electron transfer [59,103].
